# Che-1/miR-590-3p/TAZ axis sustains multiple myeloma disease

**DOI:** 10.1038/s41375-024-02168-z

**Published:** 2024-02-17

**Authors:** Tiziana Bruno, Valeria Catena, Giacomo Corleone, Clelia Cortile, Maria Chiara Cappelletto, Barbara Bellei, Francesca De Nicola, Bruno Amadio, Svitlana Gumenyuk, Francesco Marchesi, Ombretta Annibali, Giovanni Blandino, Maurizio Fanciulli, Silvia Di Agostino

**Affiliations:** 1 SAFU Laboratory, Department of Research, Advanced Diagnostics, and Technological Innovation, Translational Research Area, IRCCS Regina Elena National Cancer Institute, Rome, Italy; 2 Microbiology and Virology Unit, San Gallicano Dermatological Institute IRCCS, Rome, Italy; 3 Laboratory of Cutaneous Physiopathology and Integrated Center of Metabolomics Research, IRCCS San Gallicano Dermatological Institute, Rome, Italy; 4 Hematology and Stem Cell Transplant Unit, IRCCS Regina Elena National Cancer Institute, Rome, Italy; 5 Unit Of Hematology, Stem Cell Transplantation, Fondazione Policlinico Universitario Campus Bio-Medico, Via Alvaro del Portillo, 200, 00128 Roma, Italy; 6 Translational Oncology Research Unit, IRCCS Regina Elena National Cancer Institute, Via Elio Chianesi, 53, 00144 Rome, Italy; 7 Department of Health Sciences, Magna Græcia University of Catanzaro, 88100 Catanzaro, Italy

**Keywords:** Myeloma, Apoptosis

## To the Editor:

**Fig. 1:**
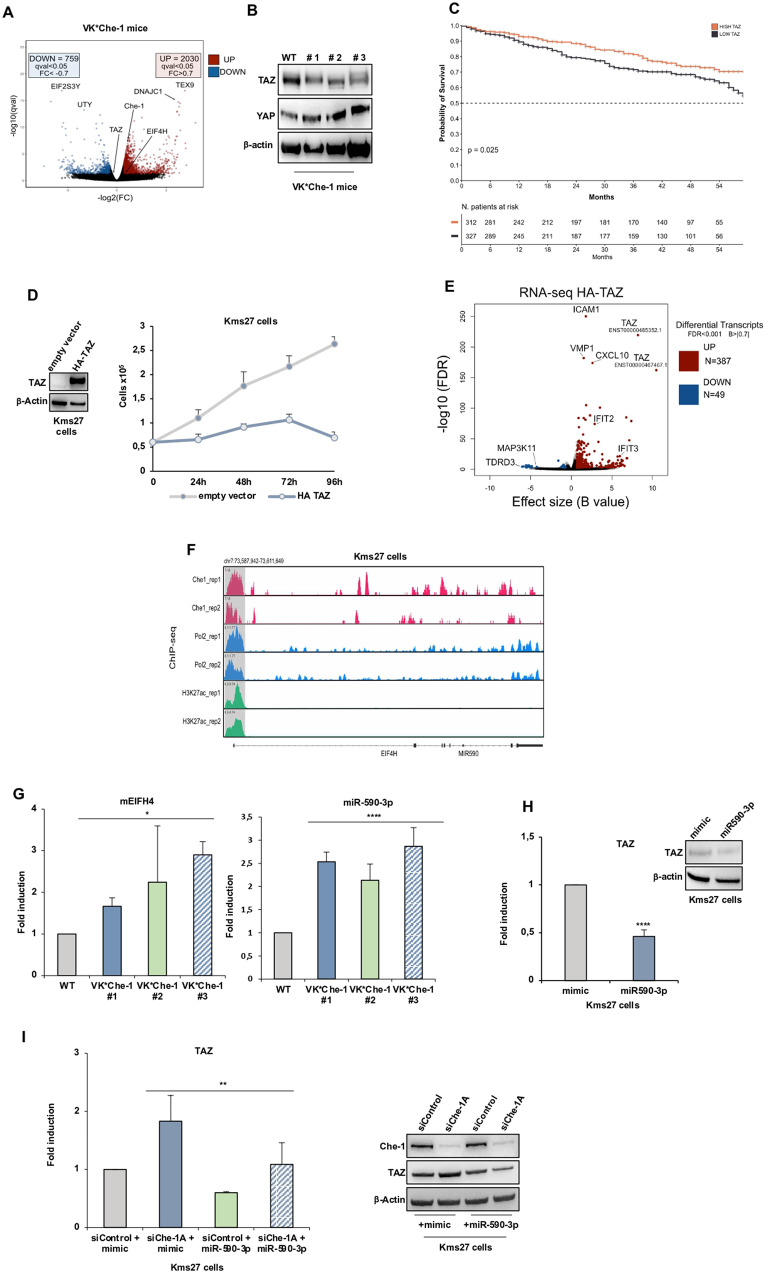
Che-1 regulates TAZ expression in MM through miR590-3p. **A** Differential analysis of Wild type c57/BL6 mice vs Vk*Che-1 mice transcriptome. Volcano plot displaying 2030 significantly upregulated (Red) and 759 downregulated (Blue) genes. x-axis: -log2(*q* value) which approximates fold change (FC) obtained from the Wald test. Y-axis: -log10 (q-value) of significant genes. **B** Total cell extracts (TCEs) from Vk*Che-1 bone marrow compared with control littermates were subjected to Western Blot analysis (WB) and probed for the indicated antibodies (Abs). **C** Kaplan–Meier survival curve of overall survival (OS) in the CoMMpass cohort (*N* = 639) with TAZ expression as unique variable. The patient cohort was divided into TAZ high (*N* = 312) (red) and TAZ low (*N* = 327) (black) by splitting the population at the TAZ median expression threshold. The patients above the TAZ median expression in our cohort were assigned to TAZ High, while patients below the median were assigned to TAZ low group. **D** Kms27 cell proliferation (10^5^) of the time course at indicated points (Right), and WB analysis (Left) with the indicated Abs after TAZ overexpression. **E** Volcano plot showing the up (Red) and down (Blue) regulated transcripts upon TAZ overexpression (HA-TAZ). X-axis: Effect size represented by the *B* value; y-axis: -log10(FDR). Differential transcripts are defined as FDR < 10-3 and *B* value > |0.7 | . **F** ChIP-seq signal on *EIF4H* promoter of Che-1, RNA Pol II and H3K27ac in Kms27 MM cells from two independent experiments. Signal enrichment scale is reported on the y-axis of the site. **G** RT-qPCR analysis of EIF4H and miR590-3p expression of bone marrow from three Vk*Che-1 mice. Values were normalized to Actin expression. Error bars represent the standard error of three different analyses. *P < 0. 05, ****P < 0.001. **H** Left: RT-qPCR analysis of TAZ expression in Kms27 MM cells transiently transfected with miR-590-3p or Control mimic. Values were normalized to Actin expression. Error bars represent the standard error of three different experiments. ****P < 0.001. Right: TCEs extracted from Kms27 MM cells transfected as above were analyzed by WB for the indicated Abs. **I** Left: RT-qPCR analysis of TAZ expression from Kms27 MM cells transiently transfected with siControl, siChe-1A, miR-590-3p or its mimic where indicated. Values were normalized to Actin expression. Error bars represent the standard error of three different experiments. **P < 0.01. Right: TCEs extracted from Kms27 MM cells transfected as above were analyzed by WB for the indicated Abs.

Multiple myeloma (MM) is a malignant disease of plasma cells that produces high levels of monoclonal immunoglobulins. This pathology involves among its clinical implications osteolytic bone disease, which represents the major cause of mortality in patients with MM [1]. YAP/TAZ co-factors belong to the HIPPO pathway, which plays an important role in epidermal and dermal development. TAZ, encoded by the *WWTR1* gene, was found to be downregulated in MM patients [2], and low TAZ expression is correlated to a decrease in osteogenic potential of mesenchymal stem cells from MM, supporting its putative involvement in bone lesions present in MM patients [3]. Che-1/AATF (Che-1) is a protein involved in various cellular pathways [4]. Recently, some studies have shown an increase in its expression levels during MM progression and its involvement in the survival of myeloma cells [5–7]. By retrieving data from RNA-seq analysis of plasma cells from a conditional Che-1 transgenic mouse model (Vk*Che-1) [5], we observed TAZ among downregulated genes in Vk*Che-1 mice (Fig. 1A). RT-qPCR and western blot analyses confirmed these results, while no changes in YAP expression were observed (Fig. 1B and Supplementary Fig. S1A). Further RT-qPCR analyses showed a decrease of mRNA expression of two TAZ target genes, *CTGF* and *CYR61* (Supplementary Fig. S1B). Conversely, the RNA-seq analysis performed in Che-1 depleted Kms27 MM cell [5] revealed an up-regulation of TAZ and its targets, but not of YAP (Supplementary Fig. S1C). These results were further confirmed at transcriptional and post translational level in Kms27 and Kms18 MM cell lines (Supplementary Fig. S1D), and similar results were also observed by depleting Che-1 expression with another specific siRNA (siChe-1B) (Supplementary Fig. S1E), but not in other cell lines of different origin (Supplementary Fig. S1F).

Previous studies have demonstrated that Che-1 is required for cell proliferation in MM [5, 6]. To evaluate whether TAZ regulation is required in this function, we performed rescue experiments, demonstrating how TAZ depletion at least partially recovers the effects of Che-1 inhibition (Supplementary Fig. S1G). Interestingly, we found a decrease of Che-1 mRNA and protein expression in MM cells overexpressing TAZ (Supplementary Fig. S1H),

Afterwards, we evaluated ~1000 MM complete transcriptomes from the CoMMpass study to evaluate the RNA levels of TAZ and YAP genes by querying patient gene expression in the context of disease evolution, using the International Staging System (ISS) as a proxy. Notably, TAZ expression significantly decreased during the progression of the MM (from stage 1 to stage 3) showing an anticorrelation with Che-1 levels (Supplementary Fig. S2A), whereas we did not observe any significant trend associated with the YAP gene (Supplementary Fig. S2B). Moreover, by using a patient cohort from Oncomine database [8], we confirmed CoMMpass results (Supplementary Fig. S2C). Accordingly, the survival analysis of 639 MM patients (CoMMpass dataset) revealed a high expression of TAZ strongly associated with a better prognosis in MM (Fig.1C) confirmed by Oncomine database [8] (Supplementary Figs. S2D, E).

To directly assess the relevance of TAZ expression in MM, we overexpressed this gene in two different MM cell lines observing in both experiments a strong reduction of cell proliferation (Fig. 1D and Supplementary Fig. S3A). Next, we performed RNA-seq analysis and unbiased GSEA in Kms27 cells overexpressing TAZ (Fig. 1E). Notably, the Hallmarks collection from the Molecular Signatures Database (MSigDB) revealed a downregulation of several pathways involved in cell proliferation (Supplementary Figs. S3B, C). Indeed, E2F1, Myc and PI3K/Akt/MTOR target genes were inhibited in TAZ overexpressing cells, conversely targets of the interferon response resulted activated (Supplementary Figs. S3D). In agreement with these results, the inhibition of Deptor or MKK6 expression strongly reduced Kms27, Kms18 or RPMI8226 cell proliferation (Supplementary Fig. S3E).

Next, we moved to identify the mechanism/s by which Che-1 regulates TAZ expression. Che-1 positively regulates gene transcription, binding promoter genes and increasing histone acetylation in MM cells [5]. Previous ChIP-seq of Che-1 experiments [5] did not show Che-1 on the *WWTR1* gene promoter, although both RNA Polymerase II and histone acetylation were present (Supplementary Fig. S4A). This evidence led us to consider an indirect way by which Che-1 could inhibit TAZ expression. Since TAZ is a target of several miRNAs [9], we hypothesized this activity as a possible mechanism. To test this hypothesis, we identified by an *in-silico* analysis the miR-590-3p targeting a highly conserved sequence contained in the 3’ UTR of TAZ (Supplementary Figs. S4B, C). Throughout a Genome Browser analysis, we observed that hsa-miR-590 is placed within the intron at 5’ of the *EIF4H* gene, a translation initiation factor. Interestingly, Che-1 ChIP-seq showed the presence of this protein on the *EIF4H* promoter together with RNA Pol II and H3K27ac (Fig. 1F), confirmed by ChIP-RT analyses in different MM cell lines (Supplementary Fig. S4D). Consistent with these results, overexpression of Che-1 was able to activate a luciferase reporter containing *EIF4H* promoter (Supplementary Fig. S4E). Notably, Vk*Che-1 mice exhibited higher levels of EIF4H mRNA and miR-590-3p when compared to their control littermates (Fig. 1G), and a decrease of both EIF4H and miR-590-3p expression was observed in Che-1 depleted MM cells (Supplementary Fig. S4F). Interestingly, miR-590-3p (and EIF4H) and TAZ expression were inversely correlated in MM cell lines (Supplementary Figs. S4G, H). Accordingly, miR-590-3p overexpression induced a reduction in TAZ levels in MM cell lines (Fig. 1H; Supplementary Fig. S4I). To assess direct mRNA regulation on TAZ, we cloned 3’-UTR of TAZ WT or mutant in a luciferase reporter vector. As shown in (Supplementary Fig. S4J).

It has been described that TAZ expression in MM is inhibited through its hypermethylation [2]. To compare the regulation of TAZ by miR-590-3p with epigenetic regulation, we treated with the demethylating agent 5-azacytidine MM cells transfected or not with an anti-sense able to downregulate miR-590-3p levels. These experiments showed that DNA demethylation has a greater impact on TAZ expression than miR inhibition, but that the combined treatments have an additive effect (Supplementary Fig. S4K). Strikingly, ectopic overexpression of miR-590-3p in MM cell lines was able to strongly counteract the increase of TAZ expression at transcriptional and protein levels produced by Che-1 depletion, (Fig. 1I and Supplementary Figs. S4L–M), thus confirming that Che-1 inhibited TAZ expression in MM by inducing EIF4H/miR-590-3p transcription.

**Fig. 2:**
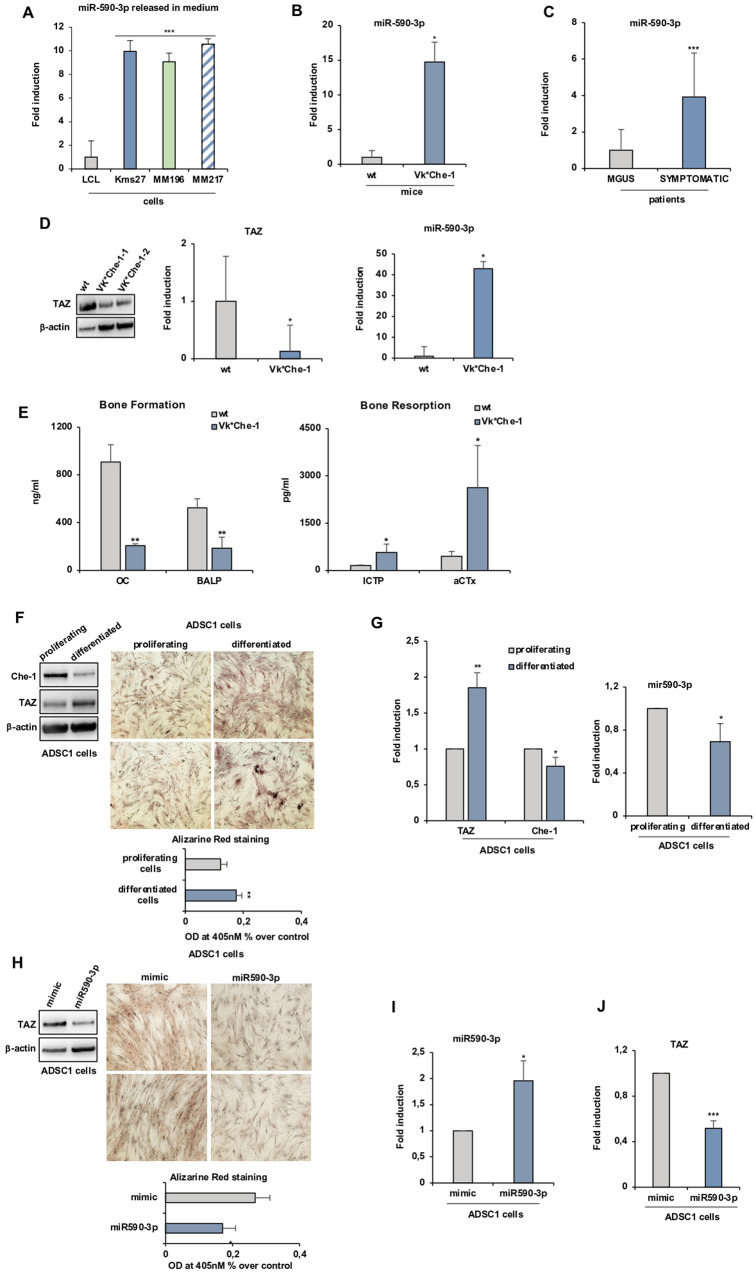
Che-1/miR590-3p inhibits osteogenic differentiation of mesenchymal stem cells. **A** RT-qPCR for estimation of the expression level of miR590-3p release in the medium of different MM cell lines compared to lymphoblastoid cells. The expression was normalized versus miRNA RNU48. Error bars represent the standard error of three different experiments. ***P < 0.005. **B** miR590-3p expression in Vk*Che-1( = 4) transgenic mice compared to their control littermates. The normalization was carried out using the mouse miRNA sno202 levels as an internal control. *P < 0.05, **P < 0.01. **C** miR590-3p expression in a cohort of patients at different stages of the disease (MGUS = 5, Symptomatic=5). The human miRNA RNU48 levels were used as an internal control. ***P < 0.005. **D** WB analysis with the indicated Abs (left) and RT-qPCR of TAZ expression (center) or miR-590-3p (right) in mesenchymal stem cells from Vk*Che-1 or control littermates bone marrow. Values were normalized to Actin expression. Error bars represent the standard error of three different experiments. *P < 0. 05. **E** ELISA assays of sera from three VK*Che-1 or three wildtype (wt) mice. C-terminal cross-linking telopeptide of type I collagen (aCTx) (competitive-ELISA principle), bone-specific alkaline phosphatase (BALP), osteocalcin (OC), and C-terminal cross-linking telopeptide of type I collagen generated by MMPs (ICTP) (sandwich-ELISA principle). *P < 0.05, **P < 0.01. **F** Left: WB analysis of TCEs from ADSC1 mesenchymal cells subjected to osteogenic differentiation and probed with the indicated Abs. Right: Two representatives Alizarin Red S staining images from ADSC1 mesenchymal cell treated as in (**F**). Bottom Bar Plot represented the measure of absorbance of stain extraction at 405 nM. Error bars represent the standard error of three different experiments **P < 0.01. **G** RT-qPCR analysis of the indicated genes (left) and miR590-3p (right) expression from ADSC1 mesenchymal cells induced as in **F**. Values were normalized to Actin expression. *P < 0.05, **P < 0.01. **H** Left: WB analysis of TCEs from ADSC1 mesenchymal cells induced for osteogenic differentiation and transiently transfected where indicated with miR-590-3p or its mimic, and probed with specific Abs. Right Two representatives Alizarin Red S staining images. Bottom The measurement of the stain from Alizarine Red experiments was performed as in (**F**). Error bars represent the standard error of three different experiments. *P < 0.05. RT-qPCR analysis for miR590-3p (**I**) and TAZ (**J**) expression from ADSC1 mesenchymal cell treated as in (**H**). Error bars represent the standard error of three different experiments. Values were normalized to Actin expression. *P < 0.05, ***P < 0.005.

Bone diseases are a major feature of MM, and an upregulation of osteoclastic activity with a concomitant decrease in osteoblasts is considered the hallmark of pathology [10]. In this context, TAZ plays an important role in osteoblastic differentiation [11] and MM cells inhibit its expression through the release of several factors in the bone marrow, such as TNF-α and FGF2 [12]. Therefore, based on the obtained results, we hypothesized that miR-590-3p could contribute to inhibit osteoblastic differentiation by downregulating the expression of TAZ. In this context, miR-590-3p was found to be released at high levels in the culture medium of several MM lines compared to the levels found in a lymphoblastoid cell line (LCL) (Fig. 2A). Moreover, we found that both Vk*Che-1 and Vk*Myc transgenic mouse model, which, through activating c-Myc oncogene in maturing B cells, induces MM transformation [13], also showed higher levels of the miR-590-3p in their bone marrow than control littermates (Fig. 2B and Supplementary Fig. S5A). These findings were further strengthened by the evaluation of the levels of miR-590-3p in the bone marrow of MGUS and symptomatic patients, finding a dramatic increase of this molecule in patients with MM (Fig. 2C). Consistent with these findings, Vk*Che-1 mesenchymal stem cells showed higher levels of miR 590-3p compared to same cells from control mice with concomitant reduced levels of TAZ (Fig. 2D). Moreover, biochemical markers analysis [14] in sera from Vk*Che-1 mice indicated a strong increase in bone resorption associated with a decrease in bone formation (Fig. 2E).

Next, we evaluated the effects of the osteogenic differentiation of two tissue-derived pluripotent mesenchymal stem cell lines, ADSC1 and ADSC2. The staining of calcium deposits by using Alizarine red S (ARS) confirmed that both these cells underwent an increase in the mineralization process (Fig. 2F and Supplementary Fig. S5C) accompanied by an upregulation of genes involved in osteoblast differentiation (Supplementary Figs. S5B, E). Interestingly, together with an increase of TAZ expression, we observed in differentiated cells a significant decrease of both Che-1 and miR590-3p levels (Fig. 2G and Supplementary Fig. 5D). Strikingly, the presence of miR590-3p in the medium of these cells strongly affected the differentiation process, decreasing calcium mineral deposits (Figs. 2H, I and Supplementary Figs. S5F, G) and a significant reduction of TAZ expression and differentiation markers (Fig. 2J and Supplementary Fig. S5G). Importantly, similar results were obtained overexpressing Che-1 in ADSC1 cells (Supplementary Fig. S5I), observing an increase of EIF4H and miR590-3p expression (Supplementary Fig. S5J), and an inhibition of TAZ expression and osteogenic differentiation markers (Supplementary Figs. S5K, L).

In conclusion, in this study, we identify a novel role of Che-1 in MM pathogenesis. Indeed, by performing experiments in cell lines, patient samples, and mice models, we found that Che-1 can negatively control TAZ expression in MM by upregulating miR-590-3p. Our results clearly demonstrated that during the differentiation of mesenchymal cells into osteoblasts, alongside the induction of TAZ and its target genes, there is also a downregulation of both Che-1 and miR-590-3p. Furthermore, we have shown that MM cells secrete this miRNA, and high levels of this molecule are found in vivo both in the bone marrow of MM mouse models and in patients. Finally, overexpression of either miR-590-3p or Che-1 significantly inhibited TAZ expression in mesenchymal stem cells along with osteoblastic differentiation.

### Supplementary information

Supplemental Information

## Data Availability

High Throughput Sequencing data of Vk*Che mice and Kms27 MM cells were retrieved from High Throughput Sequencing data (RNA-seq, ChIP-seq, ATAC-seq- identifier numbers GSE149031) previously reported in Bruno et al. [5]. High Throughput Sequencing data (RNA-seq,) were publicly deposited and are available at GEO (GSE234642). All other row data supporting the findings of this study are available at this link: https://gbox.garr.it/garrbox/f/647389073.
