# Supplementary material for: Che-1/miR-590-3p/TAZ axis sustains multiple myeloma disease
Source: Leukemia. 2024 Feb 17;38(4):877–82. doi: 10.1038/s41375-024-02168-z (PMC10997508; doi:10.1038/s41375-024-02168-z)
Supplement: Supplementary file 1 — Supplemental Information [file 41375_2024_2168_MOESM1_ESM.pdf]

Supplementary Information

23-LEU-0757R

Che-1/miR-590-3p/TAZ axis sustains multiple myeloma disease.

Bruno et al.

Table of content:

- Supplementary Material and Methods, pages 2-8
- Supplementary References, page 9
- Supplementary Table 1, pages 10-12
- Supplementary Table 2, pages 13
- Supplementary Figures S1-S5 with legends, pages 14-30

Supplementary Materials and Methods

Cell lines and cell culture

Human Multiple myeloma (MM) cell lines Kms27, Kms18, RPMI8226 and U266 were cultured in Optimem (Thermo Fisher Scientific) supplemented with 15% serum as already described [1]. Human HCT116, HeLa and BJ cell lines were cultured as already described [2, 3]. EBV-immortalized lymphoblastoid cell line (LCL) was cultured in RPMI-1640 GlutaMAX supplemented with 15% heat-inactivated fetal bovine serum, 100 U/ml penicillin, and 100 μg/ml streptomycin [4]. Mesenchymal stem cell lines (ADSC1 and ADSC2) were obtained and cultured as described in Bellei et.al [5]. All cell lines were cultured at 37°C, in a humidified atmosphere with 5% CO2. All the MM cell lines were not listed in the ICLAC database of commonly misidentified cell lines and were authenticated at The San Martino Hospital Genomics Facility using short tandem repeat DNA profiling. The presence of the Epstein-Barr virus (EBV) and Mycoplasma contamination were detected by PCR. Primer's information is provided in Supplementary Table S1.

5-Azacytidine was purchased from SIGMA (A2385).

Human specimens.

Monoclonal gammopathies of undetermined clinical significance (MGUS) and symptomatic patient samples were collected as part of routine clinical examination. The study was approved by the Regina Elena Cancer Institute Ethics Committee (CE 422/14) and written informed consent to participate in this study was provided by all subjects. Total RNA from bone marrow aspirates was extracted using TRIzol (Invitrogen-Thermofisher) and miR enrichment by miRNeasy Micro kit (Qiagen).

Mice

All the procedures involving animals and their care were approved by the Italian Ministry of Health (Prot. 130/2015-PR) and were conformed to the relevant regulatory standards in accordance with the Italian legislation. Bone marrows from Vk*Myc [6] and Vk*Che-1[1] mice were obtained from both femoral bones. Control littermates were used as negative controls. Total RNA was extracted

using TRIzol (Invitrogen-Thermofisher) and purified by miRNeasy Micro kit (Qiagen). The animal facility staff performed random treatments of every mice strain in blinding conditions.

siRNA oligonucleotides and recombinant plasmids

Transfection experiments were carried out by using the Lipofectamine 3000 transfection system (Thermo Fisher Scientific) according to the manufacturer's instructions. Cells were analyzed 48 hours after transfection, and the efficiency of transfection was determined by quantitative real time PCR (RT-qPCR) and western blotting (WB) analysis. Stealth siRNA oligonucleotides targeting Che-1 (siChe-1A, cat. n. 1299003–HSS120158 and siChe-1B, cat. n. 1299003–HSS120159), Deptor (HSS127789, HSS127790, HSS185577) and a control sequence (siControl, cat. n. 12935300) were purchased from Thermo Fisher Scientific. Sequence of two independent siRNAs mixed in an equal amount and used to interfere TAZ expression: siTAZ#1: 5′-AAAGUUCCUAAGUCAACGU-3′, siTAZ#2: 5′-AGGUACUUCCUCAAUCACA-3′. HA-TAZ expression vector was purchased from Addgene (plasmid #32839). pLKO vector containing shRNA against human MKK6 gently kind of Dr. Bossi.

3'-untranslated (3'UTR) region of TAZ, containing predicted miR-590 target site, was amplified by PCR from Kms27 cell genomic DNA. The primer sequences were as follows:
Forward 5'- AACCGCTCGAGTTCTGAGGGCATCTTGGTCCAGGAA -3'
Reverse 5'- AAAAAGCGGCCGCCTACTTTCCCGCTGGAGGGACTAAGT -3'.
3'UTR fragment was cloned in psiCHECK2 vector (Promega). The corresponding mutant construct was created by using the QuikChange site-directed mutagenesis kit (Agilent Technologies, Inc., Santa Clara, CA, USA) and following primers:
Forward 5'-CATTGTATGGATAGTGGAATTCTACTGTATGGAATAC-3'

Reverse 5'-GTATTCCATACAGTAGAATTCCACTATCCATACAATG -3'

Promoter sequence of *EIF4H* gene was amplified by PCR from Kms27 genomic DNA and cloned into PGL2-Basic vector (Promega) using following primers:

Forward 5' TACTACGGTACCCTCAAAACCCCCCAATACCCAGGC 3'

Reverse 5' TACTACAAGCTTCAATCAGGACGACGCTAAATGCGG 3'

Alizarine Red Staining

Alizarine Red S (ARS, C.I. 58005-Promocell) was prepared and stored in the dark in according to the manufacturer's instructions. Cells were fixed in 10% neutral buffered formalin for 30 min. At the end of time formalin was removed and the cells washed with distilled water. Staining with alizarin was done at room temperature in the dark for 45 minutes. Immediately after this time, the samples were washed four times with distilled. Calcium deposits were bright orange red. The stain from both uninduced and induced cultures were extracted using acetic acid and ammonium hydroxide and quantified by measuring absorbance at 405 nm. The staining images were visualized using an inverted microscope Motic AE2000 with 10X magnification.

RNA isolation and RT-qPCR

Total RNA isolation, cDNA synthesis and the RT-qPCR analyses were performed as already described [1]. The Ct was measured during the exponential amplification phase, and the amplification plots were analyzed using the software v2.0.6 (Applied Biosystems). Data are presented as the mean ± SD from three independent experiments performed in duplicate. Primer's information is provided in Supplementary Table S1.

miRNA

For mature has-miR-590-3p ectopic expression, we transfected the cells with miR-590-3p mimic (mirVana® miRNA mimic Thermo Fisher Scientific MIMAT0004801), anti-sense miR590-3p (Antagomir) (CliniSciences MNH03183), or negative mimic control (mimic) (mirVana™ miRNA Mimic, Negative Control #1 Thermo Fisher Scientific n°4464058) at the final concentration of 5 nM using Lipofectamine RNAi MAX (Thermo Fisher Scientific) according to the manufacturer's instructions. To detect human and mouse hsa-miR-590-3p, TaqMan™ MicroRNA Assay (Applied Biosystems) was used following the kit assay protocol. RNU48 (human) and sno202 (mouse) TaqMan™ Assay were used to normalize the experiments.

Western blot

Total cell extracts (TCEs) were prepared, and the proteins were resolved as previously described (29). Following blocking step in 5% non-fat-dried milk in phosphate-buffered saline (PBS)-0.1% Tween, membranes were incubated with primary antibodies overnight at 4°C. After three washes in PBS - 0.1% Tween, membranes were incubated with the appropriate HRP-linked secondary antibodies (Bio-Rad Laboratories) for 45 min at room temperature, washed with PBS-0.1% Tween and analysed by chemi-luminescence (GE Healthcare Life Science). Images were acquired and quantified using Alliance Mini HD6 system by UVITEC Ltd., Cambridge, equipped with UVI1D Software (UVI-TEC, 14-630,275). Detailed information for all antibodies is provided in Online Supplementary Table S1.

RNA-seq

Total RNA was extracted using Qiazol (Qiagen, IT), purified from DNA contamination through a DNase I (Qiagen, IT) digestion step and further enriched by Qiagen RNeasy columns for gene expression profiling (Qiagen, IT). Quantity and integrity of the extracted RNA were assessed by NanoDrop Spectrophotometer (NanoDrop Technologies, DE) and by Agilent 2100 Bioanalyzer (Agilent Technologies, CA), respectively. RNA libraries for sequencing were generated in triplicate using the same amount of RNA for each sample according to the Illumina Stranded Total RNA Prep kit with an initial ribosomal depletion step using Ribo-Zero Plus (Illumina, CA). The libraries were quantified by qPCR and sequenced in paired-end mode (2x100 bp) with NovaSeq 6000 (Illumina, CA). For each sample generated by the Illumina platform, a pre-process step for quality control was performed to assess sequence data quality and to discard low-quality reads.

Computational methods

RNA-seq analysis was run using the Kallisto v0.46.2 18 and Sleuth v0.30.019 recommended pipelines. Reads were quantified with the command kallisto quant -t 12 -b 30 and pseudoaligned to the reference genome GRCm38.96 downloaded from NCBI and successfully indexed using the

command kallisto index. Differential analysis was performed using Sleuth pipeline deploying the full model.

Luciferase assays

For *EIF4H* promoter luciferase assays, cells were transfected with 500 ng PGL2-Basic-EIF4H vector and different concentration of Myc Che-1expression vector. For TAZ 3'-UTR luciferase assays, cells were transfected with 200 ng of psiCHECK-2 vector containing WT or mutant 3'UTR of TAZ and miR-590-3p or negative control (mimic). 48 h after transfection, cells were lysed, and reporter gene expression was determined by using the Dual-luciferase reporter assay system (Promega). Total protein quantification in the extracts was determined by Bradford assay.

ELISA assays

The mouse ELISA kits were purchased from Biomatik. C-terminal cross-linking telopeptide of type I collagen (aCTx) (competitive-ELISA principle), bone-specific alkaline phosphatase (BALP), osteocalcin (OC), and C-terminal cross-linking telopeptide of type I collagen generated by MMPs (ICTP) (sandwich-ELISA principle) were employed utilizing serum obtained by bleeding Vk*Che-1 or wild-type mice without sacrifice. 100 µL of each sample was added to the micro-ELISA plate wells and combined with the antibody specific for every marker. All the procedures were conducted following the manufacturer's instructions. At the end of the assay, optical density (OD) was measured spectrophotometrically at a wavelength of 450nm. The concentration of each marker was obtained using Curve Expert 1.3 software.

Bone marrow mesenchymal stem cells isolation

Bone marrow mesenchymal stem cells (BM-MSC) were isolated as previously described by Soleimani and Nadri [7]. Briefly, mice BM were harvested under sterile conditions and cell suspension filtered and plated in 95-mm culture dishes in 1 ml of complete medium at a density of 25 10^6 cells

ml^{-1}. After 3h nonadherent cells were removed by changing the medium and replacing with fresh complete medium. After an additional 8 h of culture, the medium was replaced with 1.5 ml of fresh complete medium. This step was repeated every 8 h for up to 72 h of initial culture preventing the growth and proliferation of hematopoietic cells. after additional 3 days BM-MSC were analyzed by RT-PCR and where possible, by western blot.

Statistical analyses

Data are presented as mean of three independent experiments ± SD. Statistical analyses were performed using R software. Two-tailed Student's *t*-tests with Benjamini – Hochberg correction were performed to compare one parameter between two groups. For multiple group comparison, one-way ANOVA with Tukey HSD test was used. $P < 0.05$ was considered significant. Statistical significance is indicated by asterisks as follows: *$P < 0.05$, **$P < 0.01$, ***$P < 0.005$, ****$P < 0.001$, n.s. = not significant.

CoMMpass dataset

We downloaded the row gene counts matrix of the CoMMpass dataset (IA15 release) from the MMREF Research Gateway Portal (table name MMRF_CoMMpass_IA14a_E74GTF_Salmon_Gene_Counts). The patient cohort was filtered based on the availability of unique annotation of International Staging System (ISS) and transcriptome data and included 687 samples. Gene counts related to our selected cohort were normalized by following a multistep pipeline using edgeR functions (DGEList, estimateCommonDisp, estimateTaqwiseDisp, calcNormFactors). The matrix with normalized gene counts was expressed as transcripts per million (tpm). Gene of interest as TAZ, Che-1 and YAP were selected from the matrix and plotted by relative ISS score associated with each patient by utilizing ggpubr v.0.4.0 R package.

Survival Analysis

We carried out a survival analysis for defining the importance of TAZ in the progression of MM disease. The Overall Survival (OS) data were retrieved by downloading the table named MMRF_CoMMpass_IA15_STAND_ALONE_SURVIVAL.csv from the portal cited above. For this analysis, we removed the patients with OS greater than 65 months (n=56) from our cohort. Survival curves were obtained with the Kaplan-Meier product-limit method and compared with a log-rank test. Univariate Cox regression was computed to investigate the potential role of TAZ as a predictor of survival. The survival curve examined the patients with high level of TAZ expression versus those with low level. Patients with TAZ expression higher than its median expression in our whole cohort were included in the TAZ high group (n=312), and the other patients were collected into the TAZ low group (n=327). We used survfit and Surv functions in survival v.3.1.12 R package for the survival analysis. The Kaplan-Meier curve was generated by utilizing the function ggsurvplot of survminer v.0.4.819 R package and it had significance equal to log-rank p-value<0.001.

Supplementary references

1. Bruno T, De Nicola F, Corleone G, Catena V, Goeman F, Pallocca M*, et al.* Che-1/AATF-induced transcriptionally active chromatin promotes cell proliferation in multiple myeloma. *Blood Adv* 2020 Nov 24; **4**(22)**:** 5616-5630.

2. Catena V, Bruno T, Iezzi S, Matteoni S, Salis A, Sorino C*, et al.* CK2-mediated phosphorylation of Che-1/AATF is required for its pro-proliferative activity. *J Exp Clin Cancer Res* 2021 Jul 15; **40**(1)**:** 232.

3. Sorino C, Catena V, Bruno T, De Nicola F, Scalera S, Bossi G*, et al.* Che-1/AATF binds to RNA polymerase I machinery and sustains ribosomal RNA gene transcription. *Nucleic Acids Res* 2020 Jun 19; **48**(11)**:** 5891-5906.

4. Prodosmo A, De Amicis A, Nistico C, Gabriele M, Di Rocco G, Monteonofrio L*, et al.* p53 centrosomal localization diagnoses ataxia-telangiectasia homozygotes and heterozygotes. *J Clin Invest* 2013 Mar; **123**(3)**:** 1335-1342.

5. Bellei B, Migliano E, Tedesco M, Caputo S, Picardo M. Maximizing non-enzymatic methods for harvesting adipose-derived stem from lipoaspirate: technical considerations and clinical implications for regenerative surgery. *Sci Rep* 2017 Aug 30; **7**(1)**:** 10015.

6. Chesi M, Robbiani DF, Sebag M, Chng WJ, Affer M, Tiedemann R*, et al.* AID-dependent activation of a MYC transgene induces multiple myeloma in a conditional mouse model of post-germinal center malignancies. *Cancer Cell* 2008 Feb; **13**(2)**:** 167-180.

7. Soleimani M, Nadri S. A protocol for isolation and culture of mesenchymal stem cells from mouse bone marrow. *Nat Protoc* 2009; **4**(1)**:** 102-106.

Supplementary Table 1: Primer Index

**RT-qPCR mRNA primers**		
**Human Primers**	**Fwd/Rvs**	**Sequence 5'- 3'**
Actin	Fwd	GACAGGATGCAGAAGGAGATTACT
	Rvs	TGATCCACATCTGCTGGAAGGT
TAZ/WWTR1	Fwd	GTCACCAACAGTAGCTCAGATC
	Rvs	AGTGATTACAGCCAGGTTAGAAAG
YAP	Fwd	CACAGCATGTTCGAGCTCAT
	Rvs	GATGCTGAGCTGTGGGTGTA
CTGF	Fwd	AGGAGTGGGTGTGTGACGA
	Rvs	CCAGGCAGTTGGCTCTAATC
CYR61	Fwd	CCTTGTGGACAGCCAGTGTA
	Rvs	ACTTGGGCCGGTATTTCTTC
RUNX2	Fwd	TGGTTACTGTCATGGCGGGTA
	Rvs	TCTCAGATCGTTGAACCTTGCTA
AXIN2	Fwd	TACACTCCTTATTGGGCGATCA
	Rvs	TTGGCTACTCGTAAAGTTTTGGT
COL1A1	Fwd	GAGGGCCAAGACGAAGACATC
	Rvs	CAGATCACGTCATCGCACAAC
EIF4H	Fwd	GCATAAGGAGTGTACGGCTAGT
	Rsv	CCATCGTATGTCAAGGCTTCC
Mycoplasm	Fwd	ACTCCTACGGGAGGCAGCAGTA
	Rvs	TCGACCATCTGTCACTCTGTTAAC
Epstein-Barr virus (EBV)	Fwd	GGAACCTGGTCATCCTTTGC
	Rvs	ACGTGCATGGACCGGTTAAT
miR590-3p	Fwd	TGAGCTTATTCATAAAAGTGC
	Rvs	TGCTGCATGTTTCAATCAGA
DEPTOR	Fwd	AGCTTTGCCACCGGCTTAT
	Rvs	GGCAGAAGGGACTGTCATGAG
MKK6	Fwd	ACTGTCCATTCACTGTCACC

	Rvs	AGCAACAGAGTCCACCAAG
IFIT2	Fwd	GCGAAACAACTGCTCCATCT
	Rvs	CCAAGACATGCAAAGCCTCA
CDK2	Fwd	CCAGGAGTTACTTCTATGCCTGA
	Rvs	TTCATCCAGGGGAGGTACAAC
XPO1	Fwd	AGCAAAGAATGGCTCAAGAAG T
	Rvs	TATTCCTTCGCACTGGT CCT
CARL	Fwd	CCTGCCGTCTACTTCAAGGAG
	Rvs	GAACTTGCCGGAACTGAGAAC
ZW10	Fwd	AGCTGATTGTATGGAAGTTCCCA
	Rvs	TCTTTGTGCGATTGTTCAGTGT
NCBP2	Fwd	AAAACGCCATGCGGTACATAA
	Rvs	GCCTGCCCTCCTTAAAGCC
Laminin B1	Fwd	GCGGTGTACATCGACAAGGT
	Rvs	TCATGCGGCTTTCCATCAGT
ICAM1	Fwd	ATGCCCAGACATCTGTGTCC
	Rvs	GGGGTCTCTATGCCCAACAA
CXCL10	Fwd	AGCAGAGGAACCTCCAGTCT
	Rvs	AGGTACTCCTTGAATGCCACT
EIF4H promoter	Fwd	CTTGGGTGTCACCCGGGGACC
	Rvs	TCCGTCGGCGGGAGGTGACCT
**Mouse Primers**	**Fwd/Rvs**	**Sequence 5'- 3'**
TAZ/WWTR1	Fwd	AACAGTAGCTCAGATCCTTTCCTC
	Rvs	CCGCTCTGCCTCATCACTTGGTC
YAP	Fwd	TGAGATCCCTGATGATGTACCAC
	Rvs	TGTTGTTGTCTGATCGTTGTGA
CTGF	Fwd	GGGCCTCTTCTGCGATTTC
	Rvs	ATCCAGGCAAGTGCATTGGTA
CYR61	Fwd	CTGCGCTAAACAACTCAACGA

	Rvs	GCAGATCCCTTTCAGAGCGG
EIF4H	Fwd	AGTTCAGGGTGACATAGATGCT
	Rvs	GGGAATCCACCTCATCAAATTCT

Supplementary Table 2: Antibodies Index

Antibody	Supplier	Reference
Rabbit Polyclonal anti-Che-1	Millipore	Cat# ABC953
Mouse Monoclonal anti-β-actin	Sigma Aldrich	Cat# A5441
Rabbit anti-TAZ/WWTR1	Proteintech Group	23306-I-AP
Rabbit anti-YAP1	Invitrogen	PA1-46189
Rabbit anti-Deptor	Sigma-Aldrich	ABS222
Rabbit anti-MKK6	Cell Signaling	mAB#8550
Mouse monoclonal anti-Myc clone 9e10	Life Technologies	#13-2500

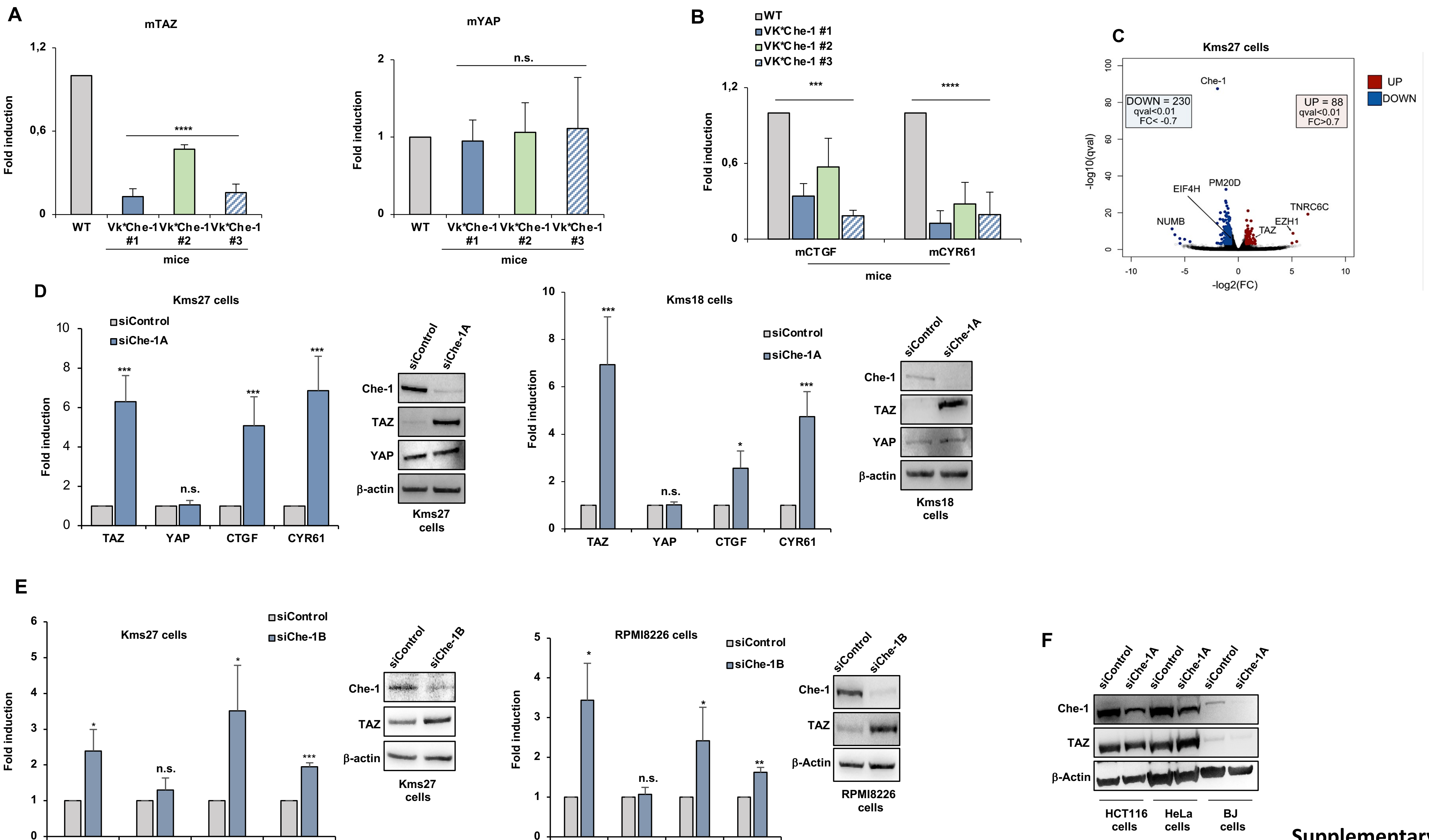

Supplementary Figure 1

G

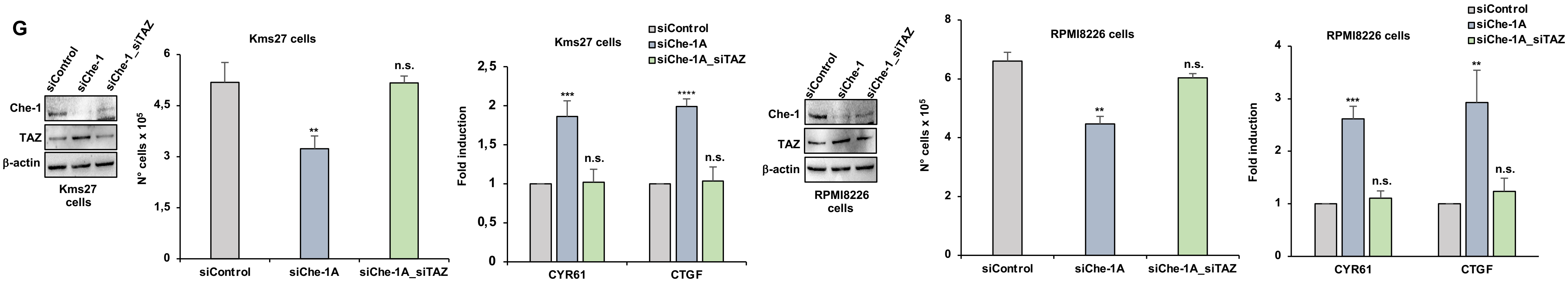

H

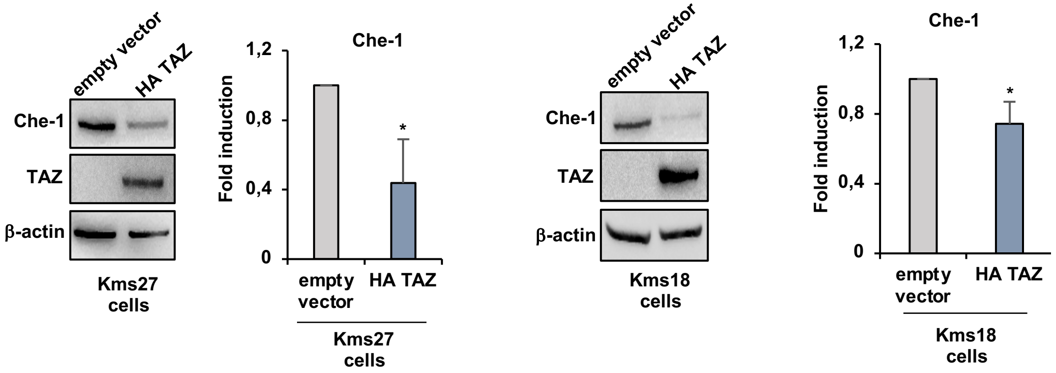

Supplementary Figure 1

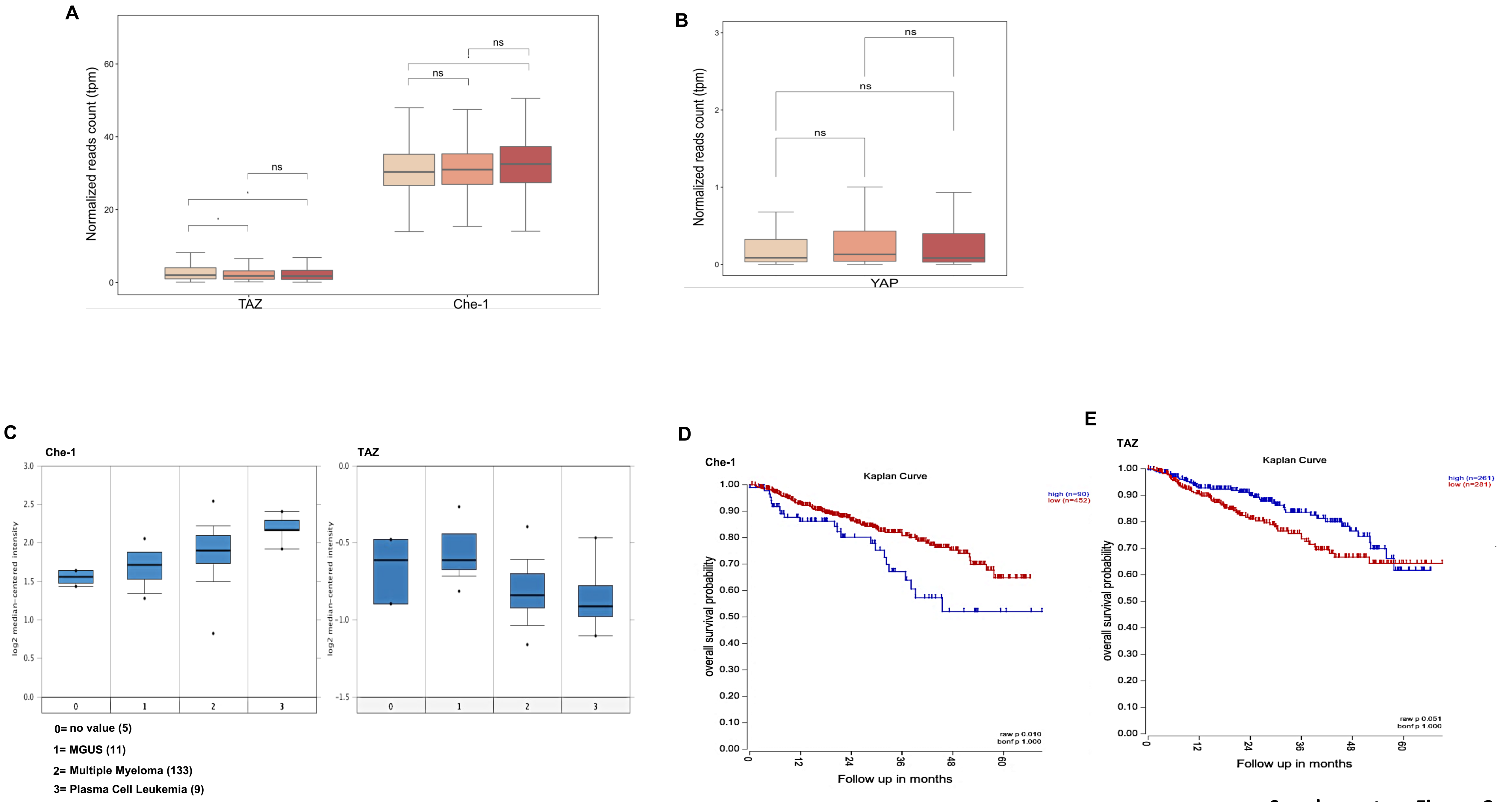

Supplementary Figure 2

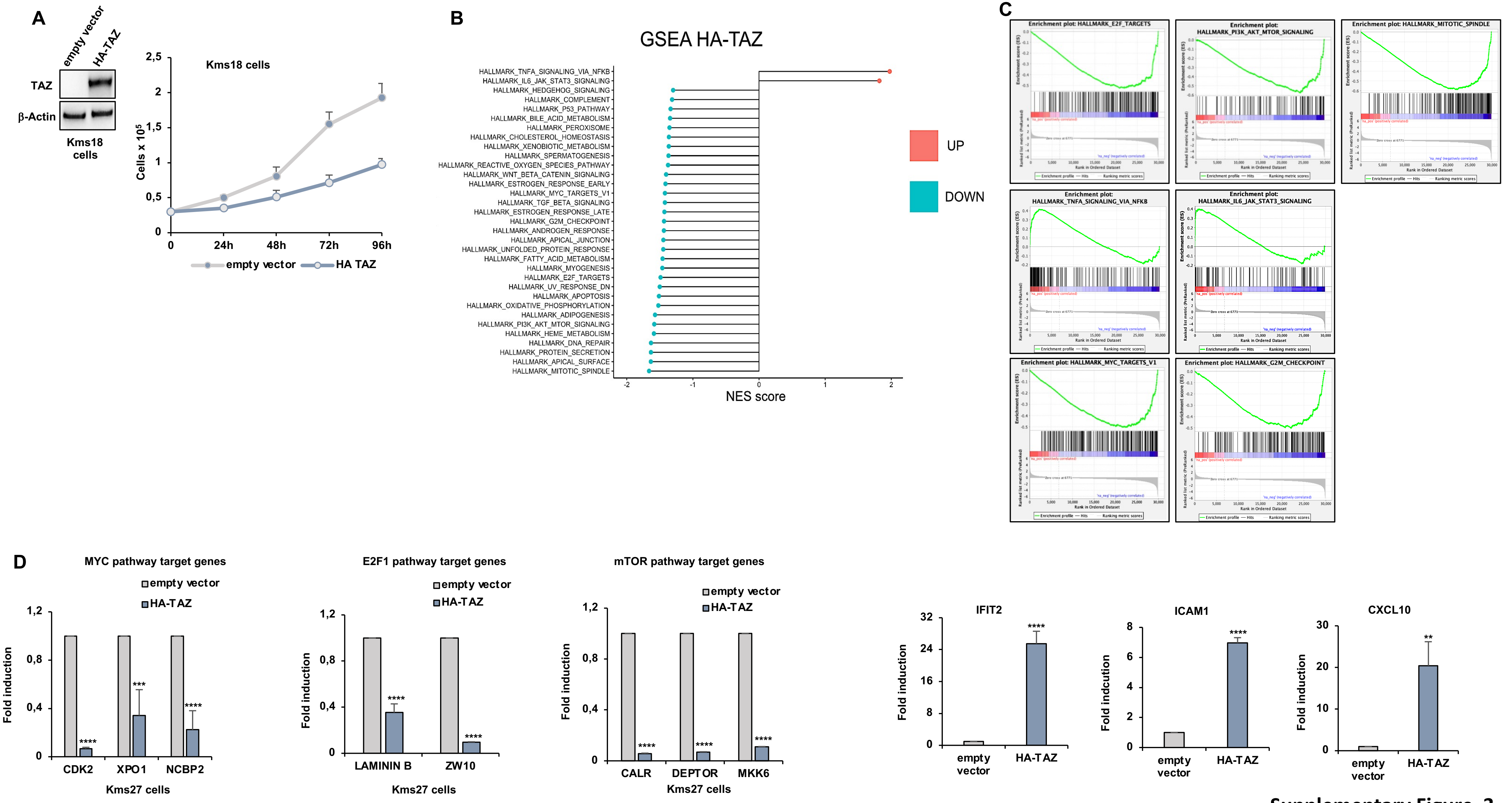

Supplementary Figure 3

E

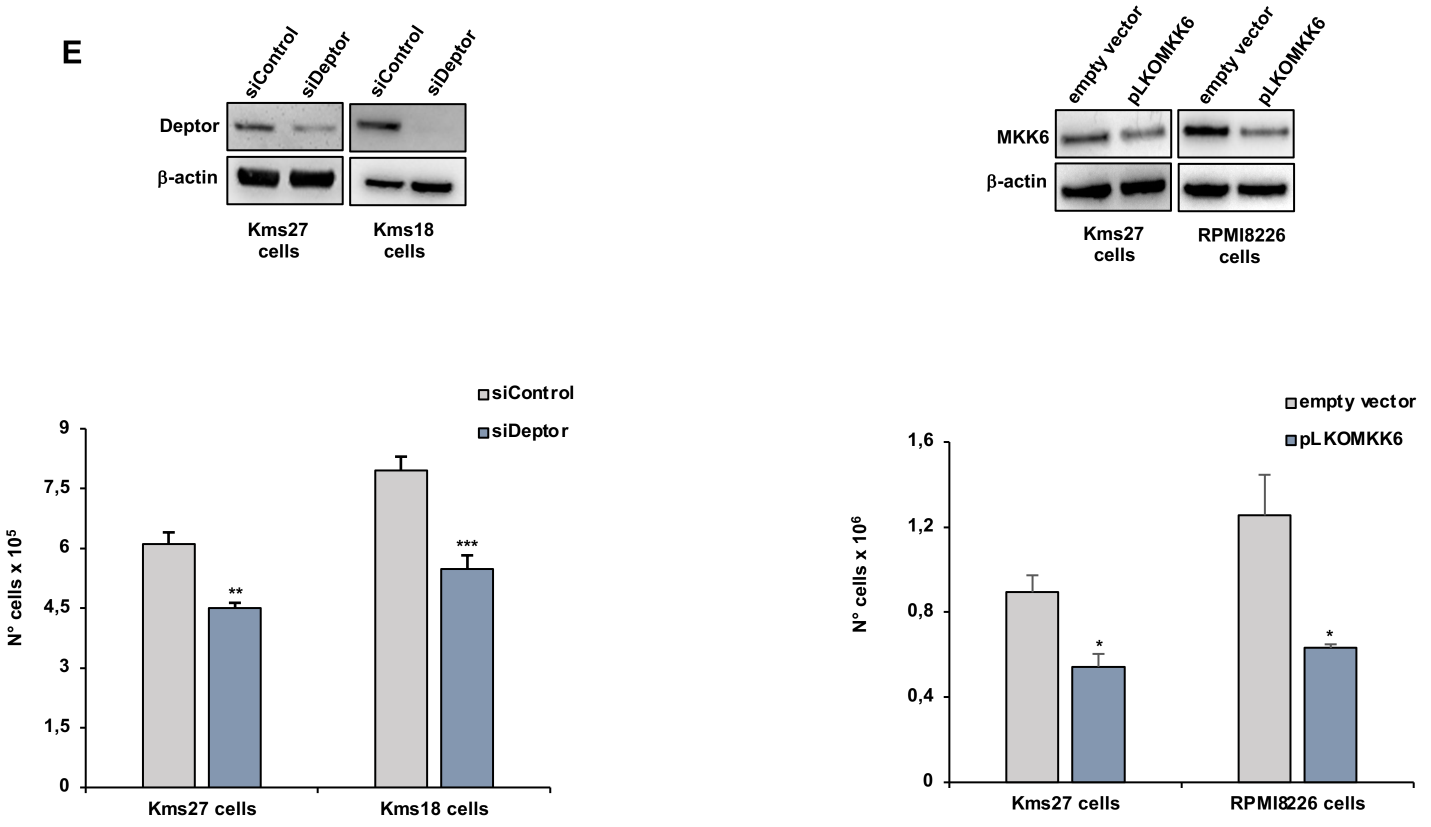

Supplementary Figure 3

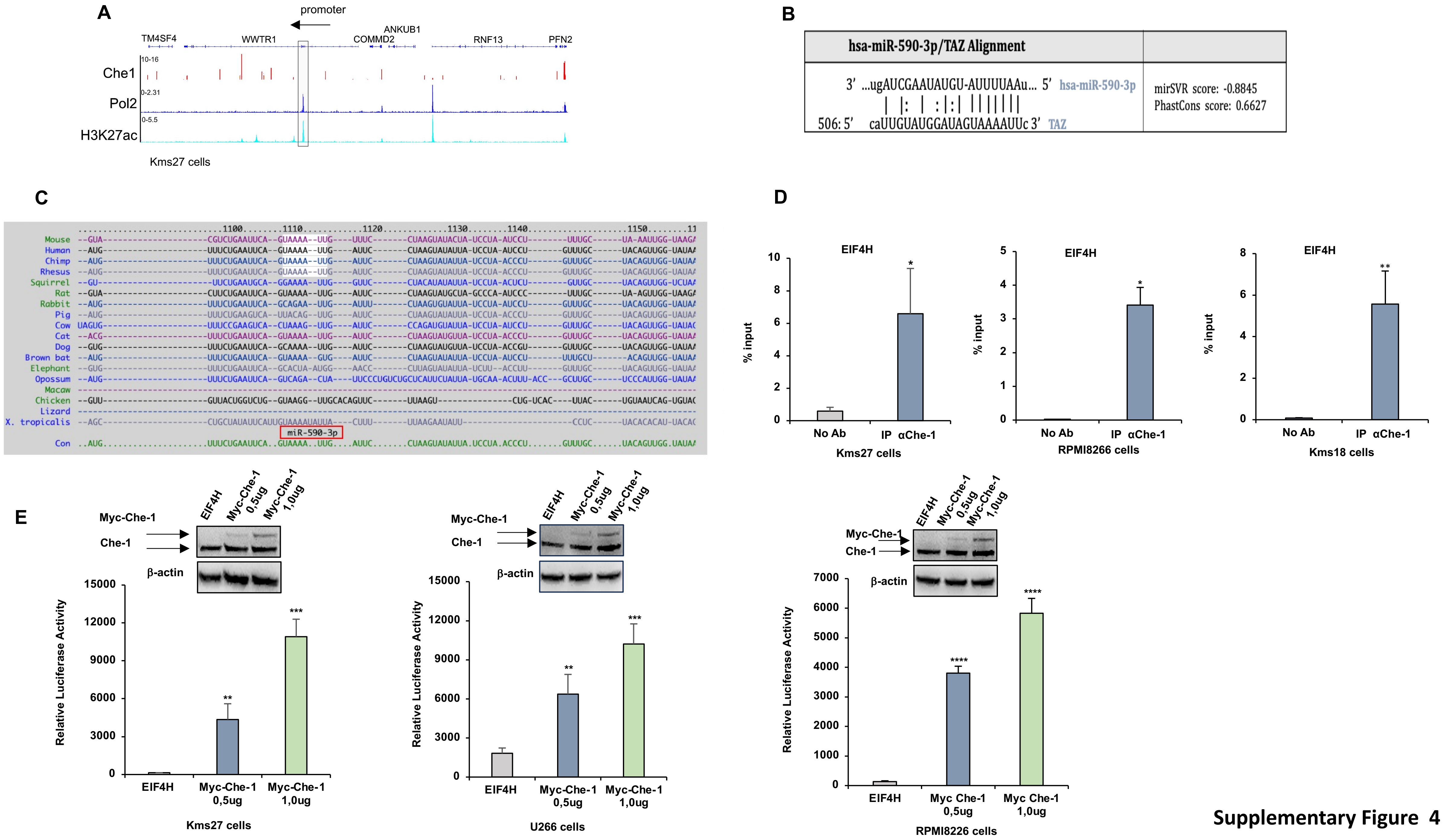

Supplementary Figure 4

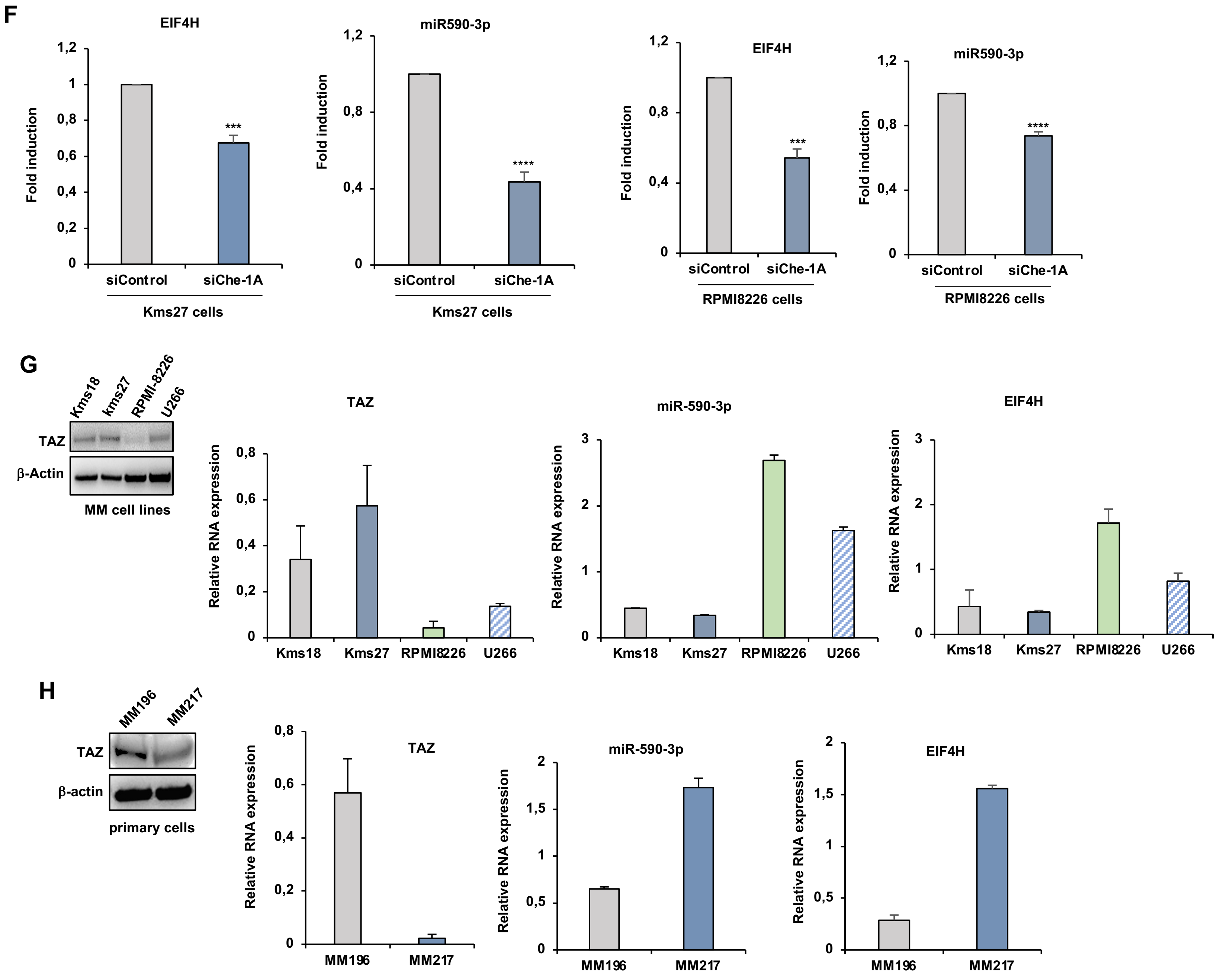

Supplementary Figure 4

I

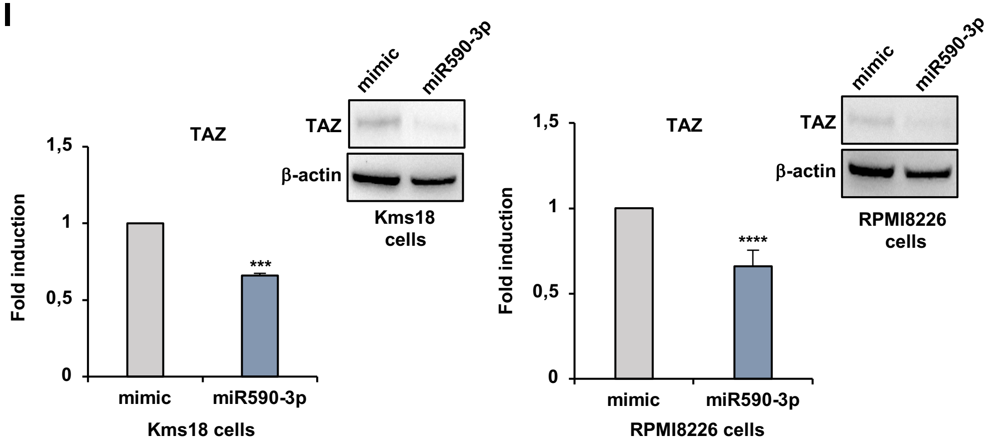

J

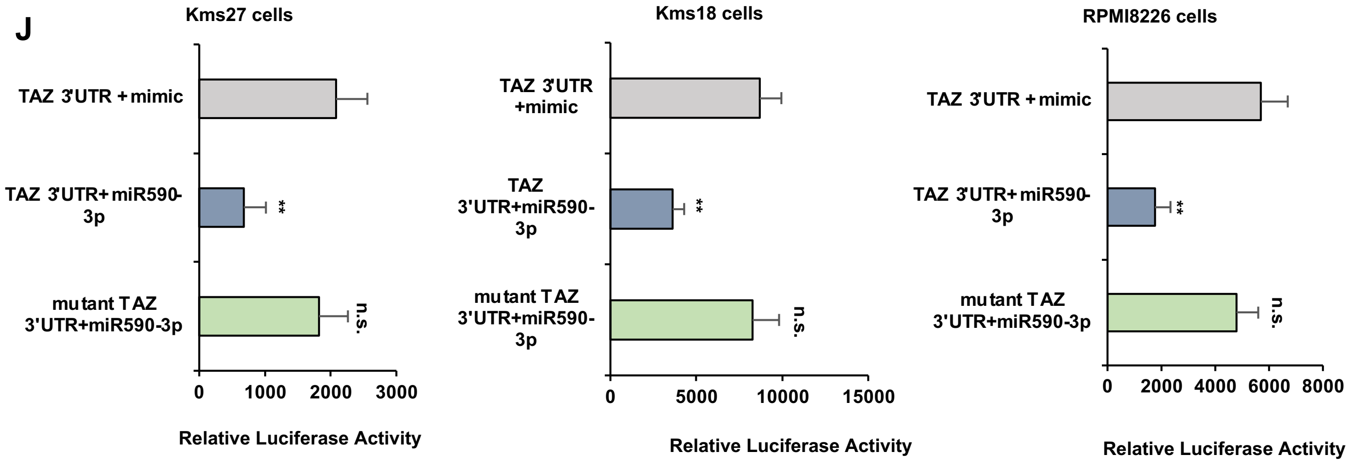

Supplementary Figure 4

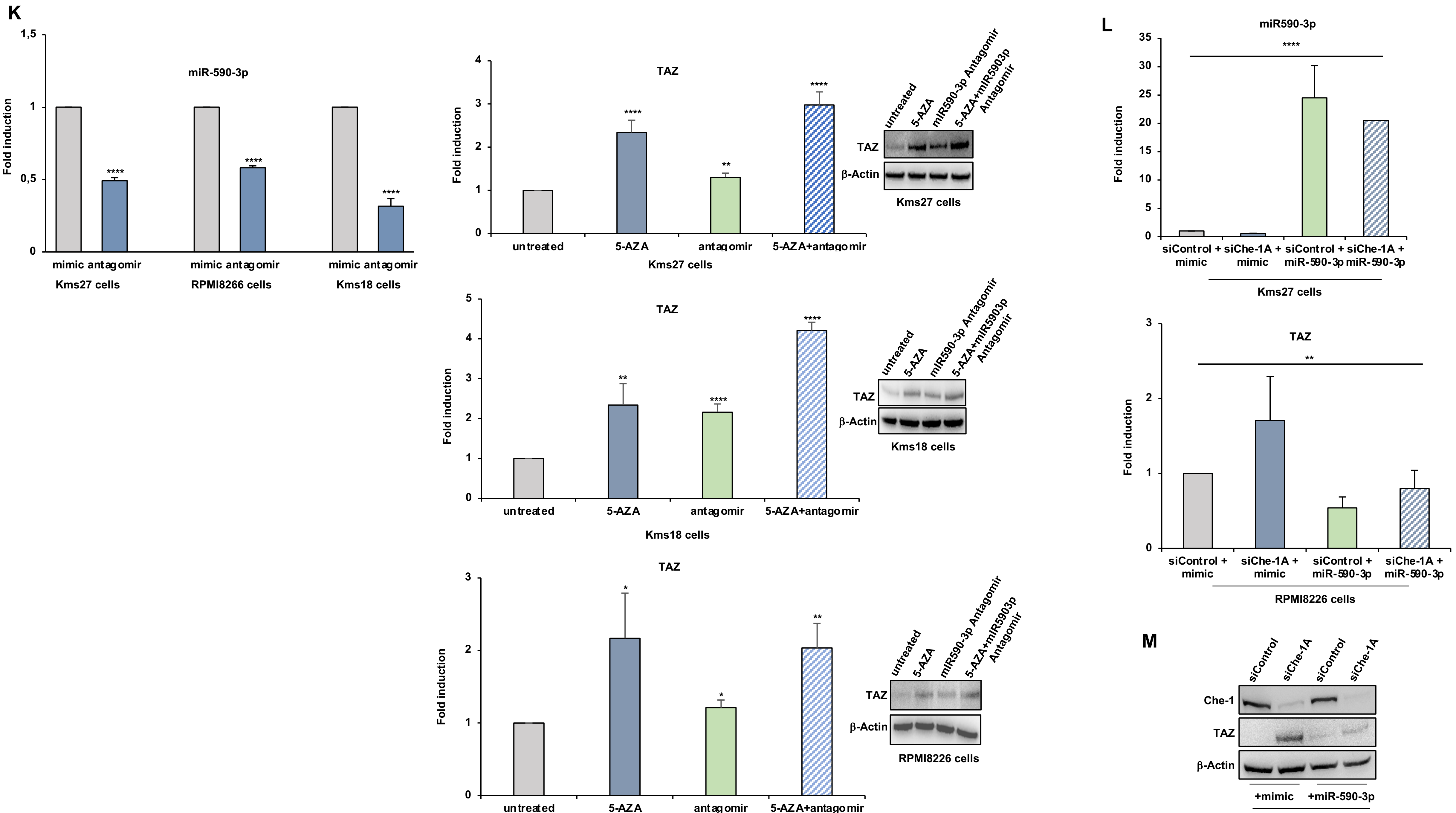

Supplementary Figure 4

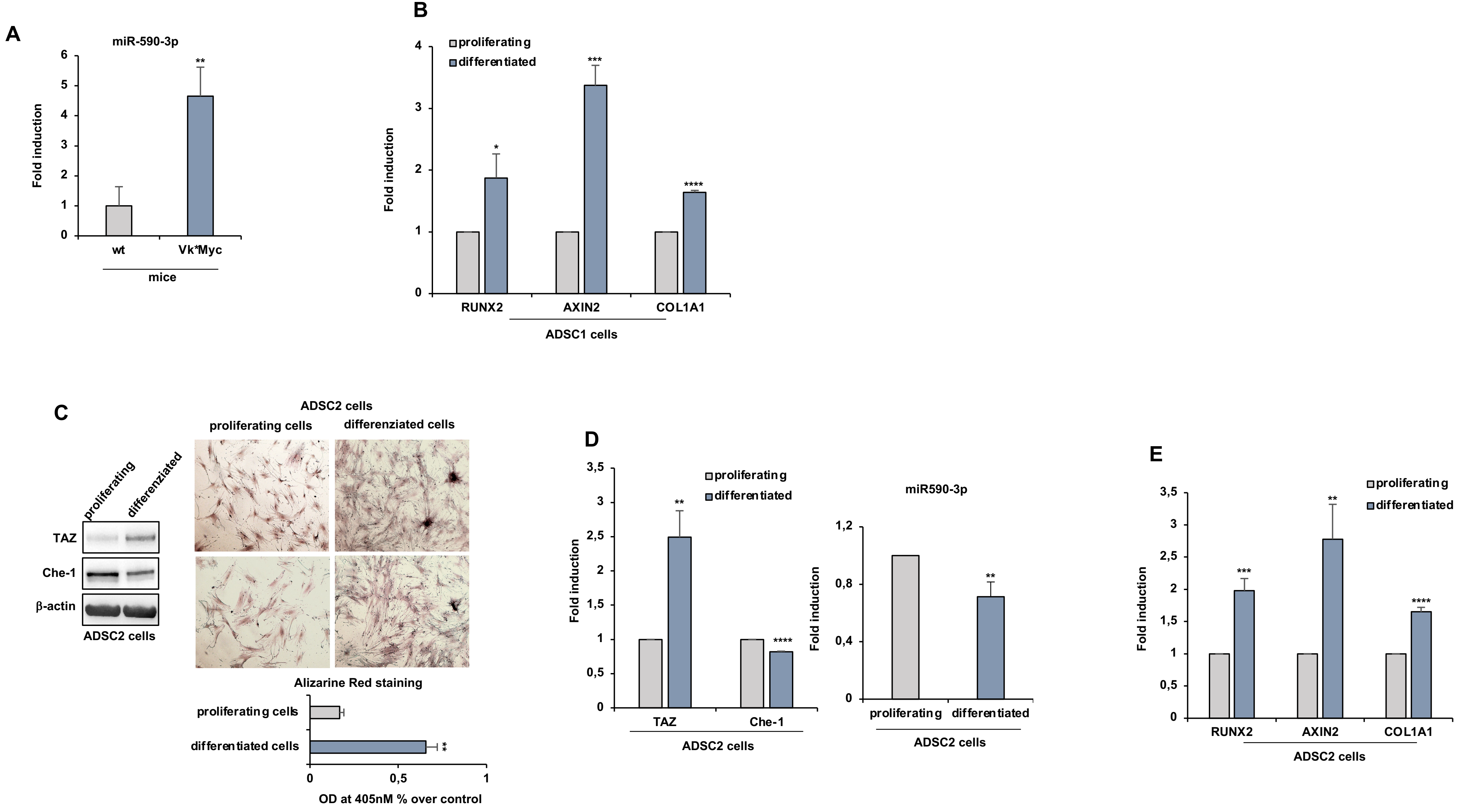

Supplementary Figure 5

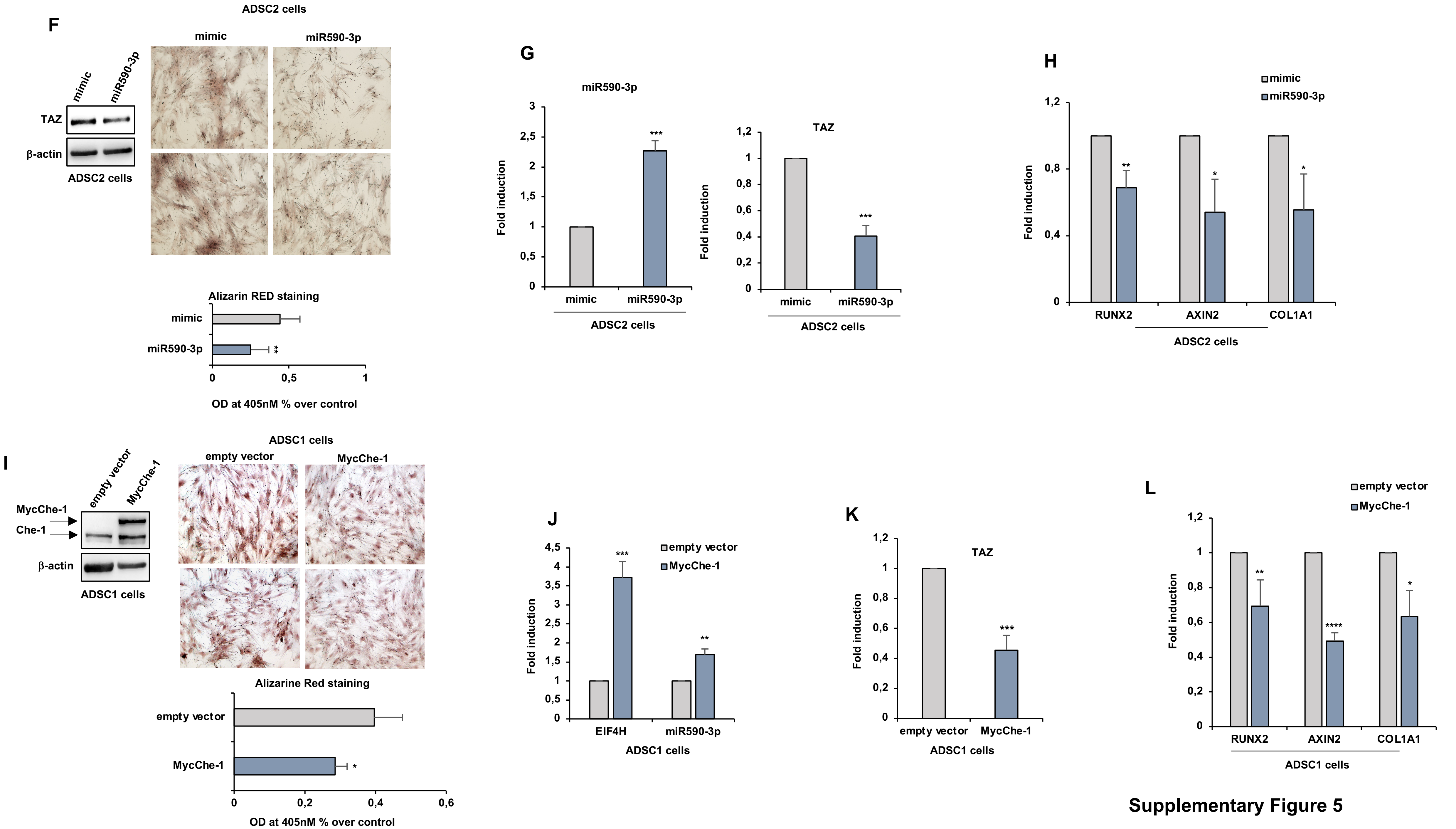

Supplementary Figure 5

Supplementary Figures Legend

Fig. S1. **Che-1 regulates TAZ expression in MM in *vivo* and in *vitro*. (A)** Quantitative real time PCR (RT–qPCR) analysis of mTAZ and mYAP levels in three Vk*Che-1 mice. Values were normalized to Actin expression. Error bars represent the standard error of three different analyses. ****P < 0.001, n.s. = not significant. (**B)** RNA was extracted from bone marrow of three Vk*Che-1 mice and analysed by RT-qPCR analysis for the indicated genes. Values were normalized to Actin expression. Error bars represent the standard error of three different analyses. ***P < 0.005, ****P < 0.001. **(C)** Differential analysis of siControl vs. siChe1 transcriptome in Kms27 MM cells. Volcano plot shows 88 significantly upregulated (Red) and 230 downregulated genes (Blue). x-axis reports the negative base-2 logarithm of fold change which is approximated to the B value coming from the statistical Wald test. y-axis reports the base-10 logarithm of Q value of significant genes. **(D) Left** Quantitative real time PCR (RT-qPCR) analysis performed in Kms27 and Kms18 Multiple Myeloma (MM) cells transiently transfected with siChe-1A or siControl and analyzed for the indicated genes. Values were normalized to Actin expression. Error bars represent the standard error of three different experiments. *P < 0.01, ***P < 0.005, n.s.= not significant. **Right** Western Blot (WB) analysis of total cell extracts (TCEs) with the indicated antibodies (Abs) of Kms27 and Kms18 MM cells transfected as described above. **(E)** RT-qPCR for the indicated genes and WB analysis with specific Abs of Kms27 and RPMI8226 MM cell lines transiently transfected with another sequence of siRNA targeting Che-1 (Che-1B). Values were normalized to Actin expression. Error bars represent the standard error of three different experiments. *P < 0.05, **P < 0.01, ***P<0.005, n.s.= not significant. **(F)** WB of TCEs from different cell lines transfected with siControl or siChe-1A and analyzed for the Abs shown. **(G) Left** WB analysis of TCEs with the indicated Abs of Kms27 or RPMI8226 MM cells transiently transfected with siControl, siChe-1 or siChe-1 and siTAZ. **Center** cell proliferation and **Right** RT-qPCR analysis for the indicated genes of Kms27 or RPMI8226 MM cells transiently transfected as above. Values were normalized to Actin expression. Error bars represent the standard

error of three different experiments. **P < 0.01, ***P<0.005, n.s.= not significant. **(H)** WB analysis for specific antibodies **(left**) and RT-qPCR **(right)** for indicated genes of Kms27 and Kms18 cells after overexpression of TAZ. Values were normalized to Actin expression. Error bars represent the standard error of three different experiments. *P < 0.01.

Supplementary Figure S2. Low TAZ expression is correlated with poor survival of MM patients. (A and B) Boxplots showing the enrichment of *TAZ*, *Che-1* (**A**), and *YAP* (**B**) gene expression by the three different ISS related to the selected CoMMpass patient cohort (N=687). The y-axis reports the normalized reads counts derived from the transcriptome of each patient and are expressed as transcripts per million (tpm). (**C**) Data from Agnelli et al. provided by the Oncomine database and reanalyzed to show expression levels of Che-1 and TAZ in normal bone marrow, MGUS, MM and plasma cell leukemia (n=158). The associated P value is shown above. Box and whisker plot show the upper and lower quartiles (25-75%) with a line at the median, whiskers extend from the 10th to the 90th percentile, and dots correspond to the minimal and maximal values. (**D and E**) Kaplan-Meier survival curve for Che-1 and TAZ expression in the Hanamura et al. cohort of MM patients (n = 542).

Supplementary Figure S3. TAZ overexpression inhibits MM cell proliferation. (**A**) WB of TCEs (**left**) and cell proliferation analysis (**right**) of Kms18 MM cells ($1x10^5$) at indicated points after TAZ overexpression. Error bars represent the standard error of three different experiments. **P < 0.01, ***P < 0.005. (**B**) Lollipop chart identifying the significant (FDR<0.05) MSigDB hallmark identified by performing the GSEA analysis. Colour scheme: red= upregulated; Blue= downregulated. X-axis represents the normalized enrichment score (NES). (**C**) GSEA profiles of the selected significant MSigDB HALLMARK. of Kms27 MM cells after overexpression of TAZ. (**D**) RT-qPCR of Kms27 overexpressing TAZ and analyzed for the indicated genes. Values were normalized to Actin expression. Error bars represent the standard error of three different experiments. **P < 0.01, ****P

< 0.001. **(E) Left** WB of TCEs with the indicated Abs **(up)** and cell proliferation **(bottom)** of Kms18 and Kms27 MM cells transiently transfected with siControl, or siDeptor. **Right** WB of TCEs with the indicated Abs **(up)** and cell proliferation **(bottom)** of Kms27 and RPMI8226 MM cells transiently transfected with empty vector or MKK6 shRNA vector (pLKOMMK6). Error bars represent the standard error of three different experiments. *P < 0.05, **P < 0.01, ***P<0.005.

Supplementary Figure S4. miR-590-3p expression Che-1-dependent affects TAZ activity in MM. (**A**) Profiles at a fixed scale of Che-1 ChIP-seq *(Red)* H3k27ac *(Light Blue)* and RNA Pol II *(Blue)* on Kms27 cells at the genomic locus of the *TAZ (WWTR1)* gene. (**B**) Sequence alignment between miR-590-3p and human TAZ. (**C**) Homology of miR-590-3p SEED sequence on *WWTR1/TAZ* gene across diverse species by TargetScan analysis. **(D)** The indicated MM cell lines were subjected to quantitative ChIP analysis (ChIP-qPCR) using anti-Che-1 antibody or control rabbit IgGs. Data are expressed as percent of input. Error bars represent the standard error of three different experiments. *P < 0.05, **P < 0.01. **(E)** The indicated MM cell lines were transiently transfected with pGL2-EIF4H luciferase reporter vector and empty vector or increasing amounts of Myc-Che-1 expression vector. **Left** WB of TCEs with the indicated Abs. **Right** luciferase activity. Error bars represent the standard error of three different experiments. **P < 0.01, ***P<0.005, ****P < 0.001. n.s.= not significant. **(F)** RT-qPCR for the endogenous expression of EIF4H (**left**) and miR-590-3p (**right**) in Kms27 and RPMI8226 MM cells depleted or not for Che-1A. Values were normalized to Actin expression. Error bars represent the standard error of three different experiments. ***P < 0.005, ****P < 0.001. **(G and H) Left** WB analysis with the indicated Abs of TCE from four MM cell lines **(G),** or two primary MM cell lines **(H). Right** RT-qPCR for the indicated genes of four MM cell lines **(G),** or two primary MM cell lines **(H).** Error bars represent the standard error of three different experiments. **(I) Left** TAZ expression analysis by RT-qPCR of the indicated MM cells lines transiently transfected with negative control (mimic) or miR-590-3p. **Right** WB analysis with the indicated Abs of TCE from MM cell lines transiently transfected as above. **(J)** Luciferase activity of

TAZ 3'-UTR or mutant TAZ 3'UTR was measured in the indicated MM cell lines transiently transfected with negative control (mimic) or miR-590-3p. Error bars represent the standard error of three different experiments. **P < 0.01, n.s.= not significant. **(K) Left** RT-qPCR analysis of miR-590-3p in the indicated MM cell lines transiently transfected with negative control (mimic) or miR-590-3p antisense (antagomir). Values were normalized to RNU48 expression. Error bars represent the standard error of three different experiments. ****P < 0.001. **Center** TAZ expression was evaluated by RT-qPCR in the indicated MM cell lines treated or not with 5-Azacytidine (5-AZA) and transiently transfected or not with antagomir. Error bars represent the standard error of three different experiments. *P < 0.05, **P < 0.01, ****P < 0.001. **Right** WB with the indicated Abs of TCE from MM cell lines treated as above. (**L**) **Up** RT-qPCR for ectopic expression of miR-590-3p in Kms27 MM cells. Values were normalized to Actin expression. Error bars represent the standard error of three different experiments. ****P < 0.001. **Bottom** RPMI8226 MM cells transiently transfected with siControl, siChe-1A, miR-590-3p or its mimic where indicated. Values were normalized to Actin expression. Error bars represent the standard error of three different experiments. **P < 0.01. (**M**) WB of TCEs from RPMI8226 MM cell line transfected as in **L** and analyzed for the indicate Abs.

Supplementary Figure S5. miR590-3p negatively influences osteogenic differentiation in primary mesenchymal cell lines.

(A) miR590-3p expression in Vk*Myc (=4) transgenic mice compared to their control littermates. The normalization was carried out using the mouse miRNA sno202 levels as an internal control. *P<0.05, **P<0.01. (**B)** The expression of *RUNX2*, *AXIN2* and *COL1A1* genes was evaluated by qRT-PCR assays in ADSC1 mesenchymal cells induced and not induced for osteogenic differentiation. Error bars represent the standard error of three different experiments. Values were normalized to Actin expression. *P<0.05, ***P<0.005, ****P < 0.001. (**C**) **Left**: WB analysis of TCEs of ADSC2 mesenchymal cell line induced or not for osteogenic differentiation and analyzed for specific antibodies. **Right**: Two representative images of Alizarine Red S staining from ADSC2

mesenchymal cell line induced or not. **Bottom**: Bar Plot represented the measure of absorbance of Alizarine Red stain extraction at 405nM. Data is the mean of three different experiments. **P<0.01. **(D)** RT-qPCR of the expression of TAZ, Che-1 (**left**) and miR590-3p (**right**) after the induction of the osteogenic program. Values were normalized to Actin expression. Error bars represent the standard error of three different experiments. **P < 0.01, ****P < 0.001. (**E**) Evaluation of the expression of the osteogenic differentiation markers *RUNX2, AXIN2 and COL1A1* of ADSC2 mesenchymal cell line induced or not as in **C**. Values were normalized to Actin expression. Error bars represent the standard error of three different experiments. **P < 0.01, ***P < 0.005, ****P < 0.001. **(F) Left**: WB analysis of TAZ expression in ADSC2 mesenchymal cell line overexpressing ectopic miR590-3p and subjected to osteogenic differentiation. **Right**: Two representative Alizarine Red S staining images of ADSC2 mesenchymal cell line. **Bottom**: Bar Plot represented the measure of absorbance of Alizarine Red stain extraction at 405nM. Error bars represent the standard error of three different experiments. **P<0.01. (**G**) RT-qPCR analysis for miR590-3p ectopic expression (**left**) and TAZ (**right**) in ADSC2 mesenchymal cell line induced or not as in **F**. Values were normalized to Actin expression. Error bars represent the standard error of three different experiments. ***P < 0.005. (**H**) Evaluation of the expression of the indicated genes of ADSC2 mesenchymal cell treated as in **F.** Values were normalized to Actin expression. Error bars represent the standard error of three different experiments. *P < 0.05, **P < 0.01, ***P < 0.005. (**I**) **Left**: WB analysis of TCEs of ADSC1 mesenchymal cell line subjected to osteogenic differentiation and transiently transfected with Myc Che-1 or empty vector and analyzed for the indicated Abs. **Right**: Two representatives Alizarin Red S staining images from ADSC1 mesenchymal cell treated as in **I. Bottom**: Bar Plot represented the measure of absorbance of stain extraction at 405nM. Error bars represent the standard error of three different experiments. *P<0.05. (**J and K**) RT-qPCR analysis of the indicated genes and for miR590-3p ectopic expression from ADSC1 mesenchymal cells transfected as in **I.** Values were normalized to Actin expression. Error bars represent the standard error of three different experiments. **P < 0.01, ***P<0.005. (**L**) RT-qPCR of RUNX2, AXIN2 and COL1A1 expression in ADSC1

mesenchymal cells treated as in **I**. Values were normalized to Actin expression. Error bars represent the standard error of three different experiments. *P < 0.05, **P < 0.01, ****P < 0.001.
